# The effect of the educational intervention based on the protection motivation theory (PMT) on the promotion of drowning prevention behaviors caused by falling vehicles in natural waters

**Published:** 2024-10

**Authors:** Nasibe Farmani Qasabe, Gholamreza Garmaroudi, Ehsan Kazemnezhad Leyli, Hassan Farrahi, Atoosa Rahbar

**Affiliations:** ^*a*^ Department of Health Education and Promotion, Faculty of Health, Tehran University of Medical Sciences, Tehran, Iran.; ^*b*^ Department of Biostatistics, School of Health, Road Trauma Research Center, Guilan University of Medical Sciences, Rasht, Iran.; ^*c*^ Department of Psychiatry, School of Medicine, Guilan University of Medical Sciences, Rasht, Iran.; ^*d*^ Head of Unit Health Education and Promotion, assistance Health, Guilan University of Medical Sciences, Rasht, Iran.

## Abstract

**Background:**

One of the important issues that has received less attention regarding drowning is drowning caused by vehicles falling into natural waters. This research aims to investigate the effect of an educational intervention based on the theory of protection motivation (PMT) on the promotion of drowning prevention behaviors caused by vehicle crashes in natural waters.

**Methods:**

In this study, 200 middle-aged men and women living in the western villages of Guilan province (100 people in the test group) and (100 people in the control group) participated in 2023. The research tool is a researcher-made questionnaire. It included two parts, which were evaluated in the first part (demographic variables) and in the second part (constructs of conservation motivation theory). Before the implementing the intervention, a pre-test was conducted and after analyzing the results, an appropriate educational intervention was designed and conducted only in the test group. The intervention results were analyzed two months later with SPSS software Version 26. The statistical tests used included independent t-test, paired t-test, chi-square, Pearson's linear correlation coefficient and regression analysis.

**Results:**

There was a significant difference between the average scores of all constructs of the PMT in the test and control groups. In all cases, the test group was more favorable than the control group (P less than 0.05). There was a significant difference between all the constructs before and after the educational intervention (P less than 0.05). However, in the control group, there was no significant difference between the average scores of all structures before and after the intervention (P less than 0.05).

**Conclusions:**

The results of this study showed that the protection motivation theory was one of the appropriate theories of health education for designing educational programs in order to promote preventive behaviors of drowning caused by falling vehicles in natural waters.

**Keywords:**

Educational intervention, Protection motivation theory, Prevention, Drowning, Natural waters

